# Supplementary material for: Transient Complexity of E. coli Lipidome Is Explained by Fatty Acyl Synthesis and Cyclopropanation
Source: Metabolites. 2022 Aug 24;12(9):784. doi: 10.3390/metabo12090784 (PMC9500627; doi:10.3390/metabo12090784)
Supplement: Supplementary file 1 [file metabolites-12-00784-s001.zip › metabolites-1877287-supplementary.pdf]

Supplementary information for:

Transient complexity of *E. coli* lipidome is explained by fatty acyl synthesis and cyclopropanation

Nikolay V. Berezhnoy[1,2], Amaury Cazenave-Gassiot[3,4], Liang Gao[3,4], Juat Chin Foo[4], Shanshan Ji[4], Viduthalai Rasheedkhan Regina[1], Pui Khee Peggy Yap[1], Markus R. Wenk[3], Staffan Kjelleberg[1], Thomas William Seviour[1,5], Jamie Hinks[1]

[1]Singapore Centre for Environmental Life Sciences Engineering, Nanyang Technological University, 60 Nanyang Drive, SBS-01n-27, Singapore, 637552.

[2]Singapore-HUJ Alliance for Research and Enterprise, Molecular Mechanisms of Inflammatory Diseases Program, and Department of Microbiology and Immunology, Yong Loo Lin School of Medicine, National University of Singapore, Singapore.

[3]Department of Biochemistry and Precision medicine Translational Research Program, Yong Loo Lin School of Medicine, NUS, Singapore.

[4]Singapore Lipidomics Incubator, Life Sciences Institute, National University of Singapore, Singapore.

[5]Aarhus University Centre for Water Technology, Department of Biological and Chemical Engineering, Universitetsbyen 36, 8000 Aarhus, Denmark

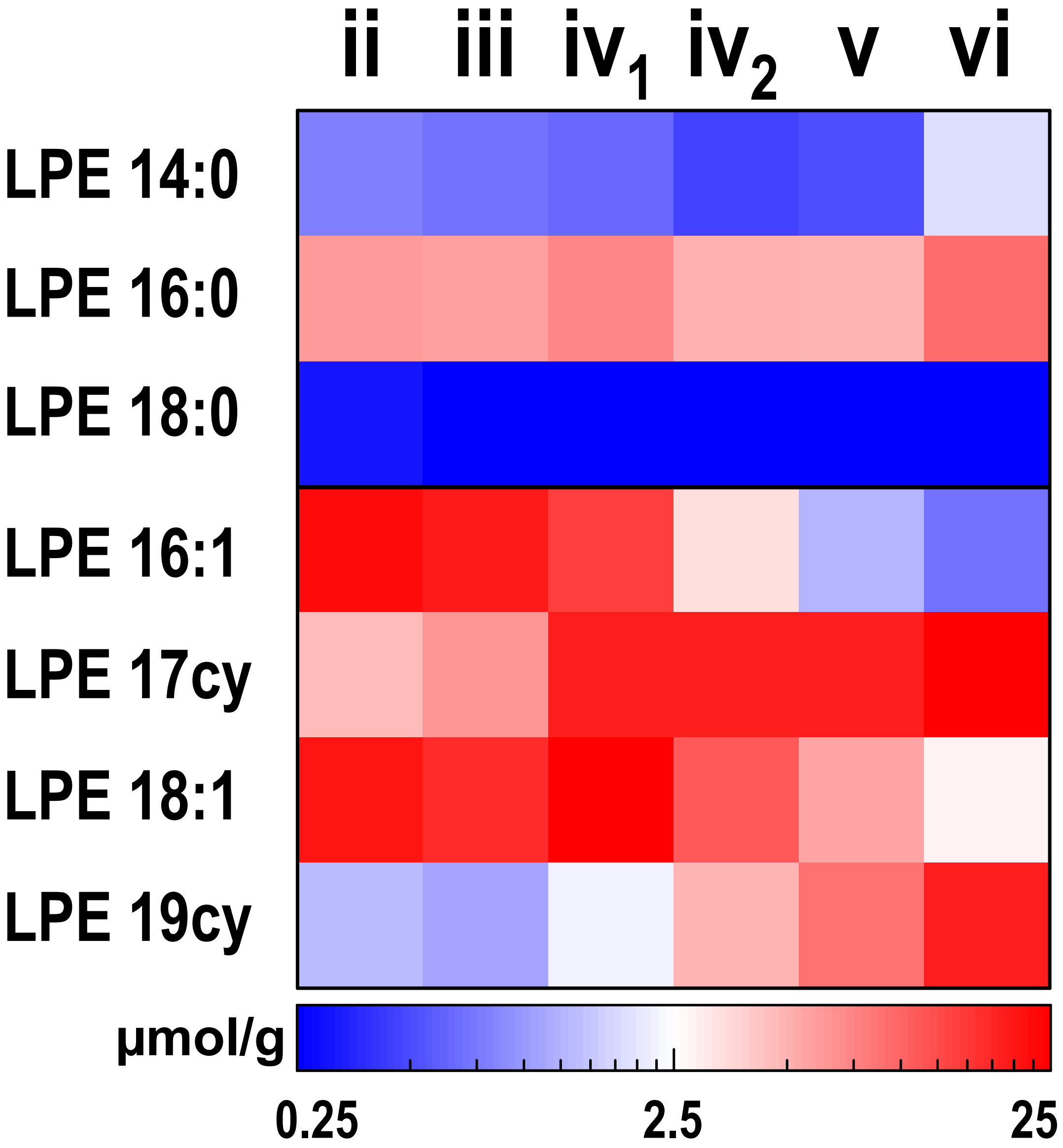

Figure S1. Abundance dynamics of LPE molecular species in cultures of BW25113 strain. Growth phases are indicated by roman numbers (abscissa) using notation introduced in **Figure 2**, where $iv_{1\text{-}2}$ correspond to samples from the deceleration phase. Molecules are grouped based on the number of DBEs (ordinate). Head group and FA chain abundance values for the heat map plotting are provided in **Supplementary Materials.**

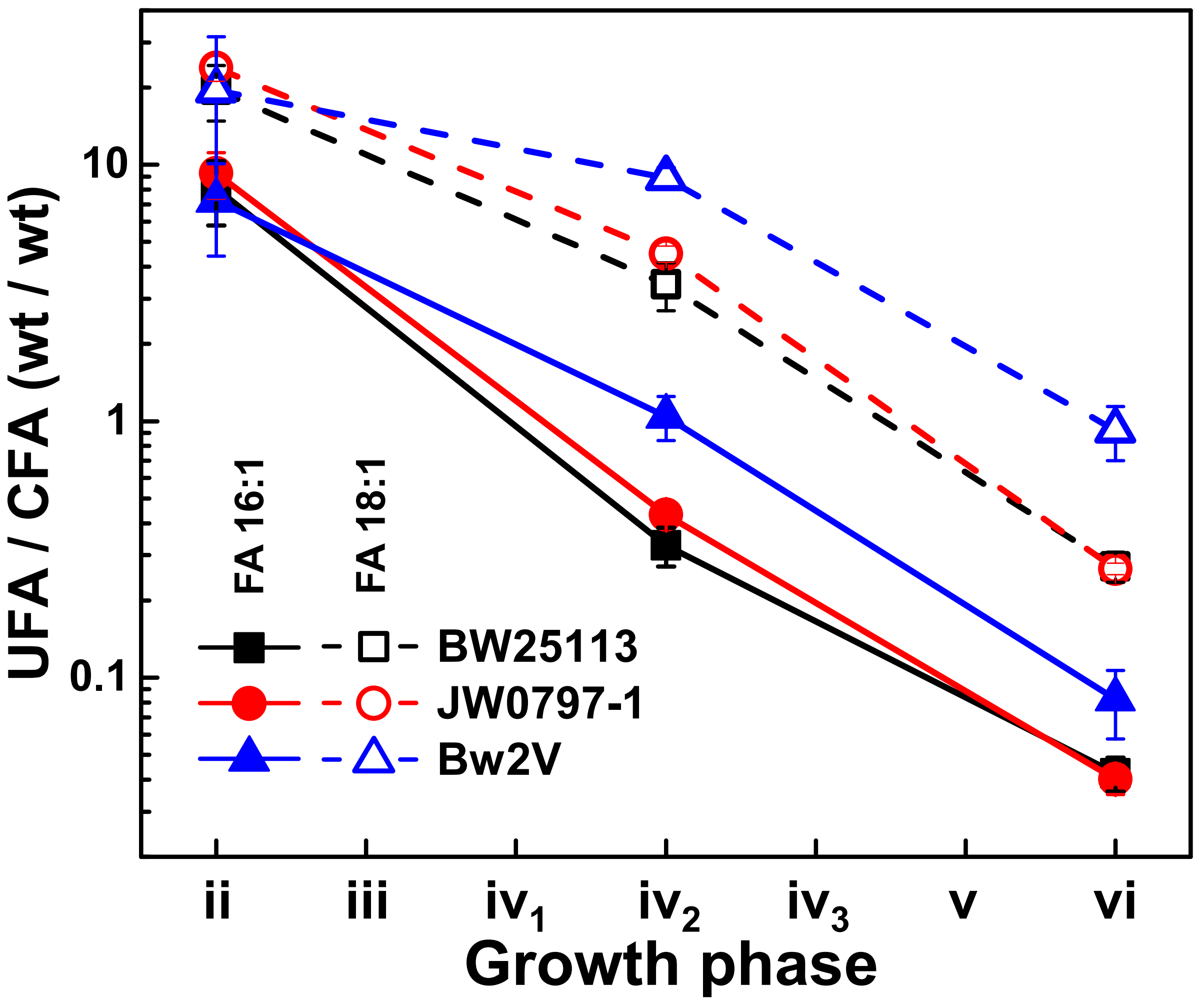

Figure S2. Ratios of unsaturated FA to cyclopropanated FA obtained from GC-MS in *E. coli* showed a gradual decrease, indicating accumulation of cyclopropanated FAs during culture growth. FA 16:1 (indicated by solid symbols) were cyclopropanated before FA 18:1 (indicated by empty symbols), in agreement with LC-MS results. FA abundance values for the graph generation are provided in **Supplementary Materials**.

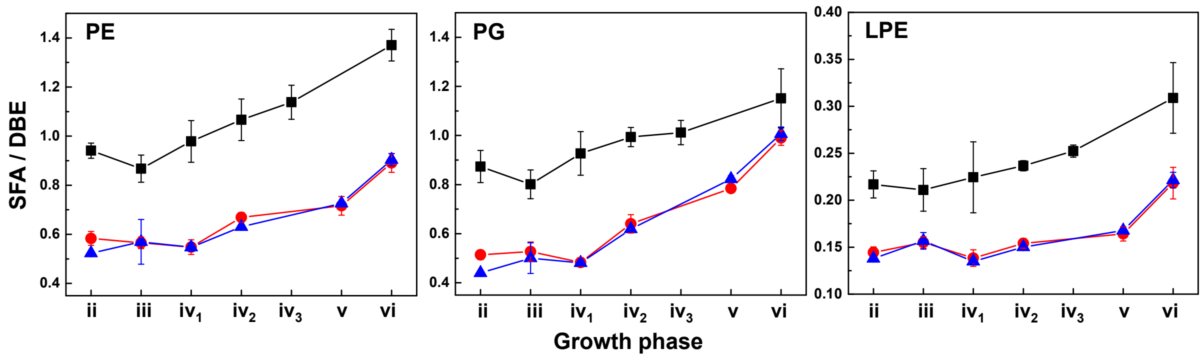

Figure S3. Ratios of SFA to DBE chains in GP classes during culture growth. BW25113 (red) and JW0797-1 (blue) strains show lower SFA/DBE ratios, in comparison with Bw2V (black) strain grown in the presence of antibiotics. The lipid abundance values for the graph generation are provided in **Supplementary Materials**.

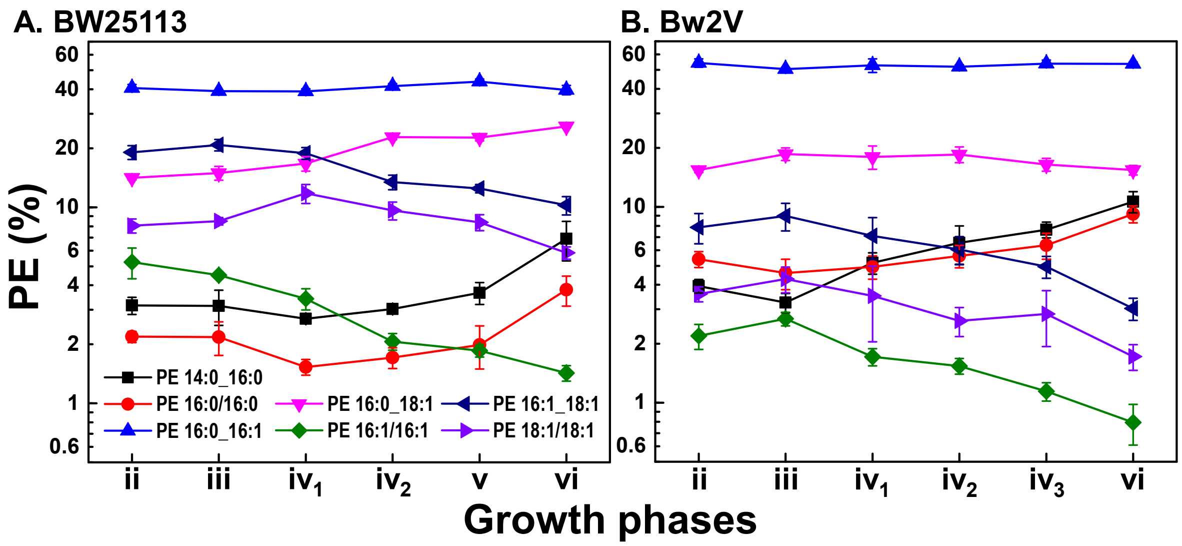

Figure S4. Abundance dynamics of the major PE species during culture growth of BW25113 (**A**) and Bw2V (**B**) strains. Species related by cyclopropanation were combined to display only the variation related to GP synthesis. For example, PE 16:0_16:1 on the graph represents a sum of PE 16:0_16:1 and PE 16:0_17cy. Therefore, the cyclopropanation-induced decrease in abundance of the former, and increase of the latter does not affect the combined abundance.

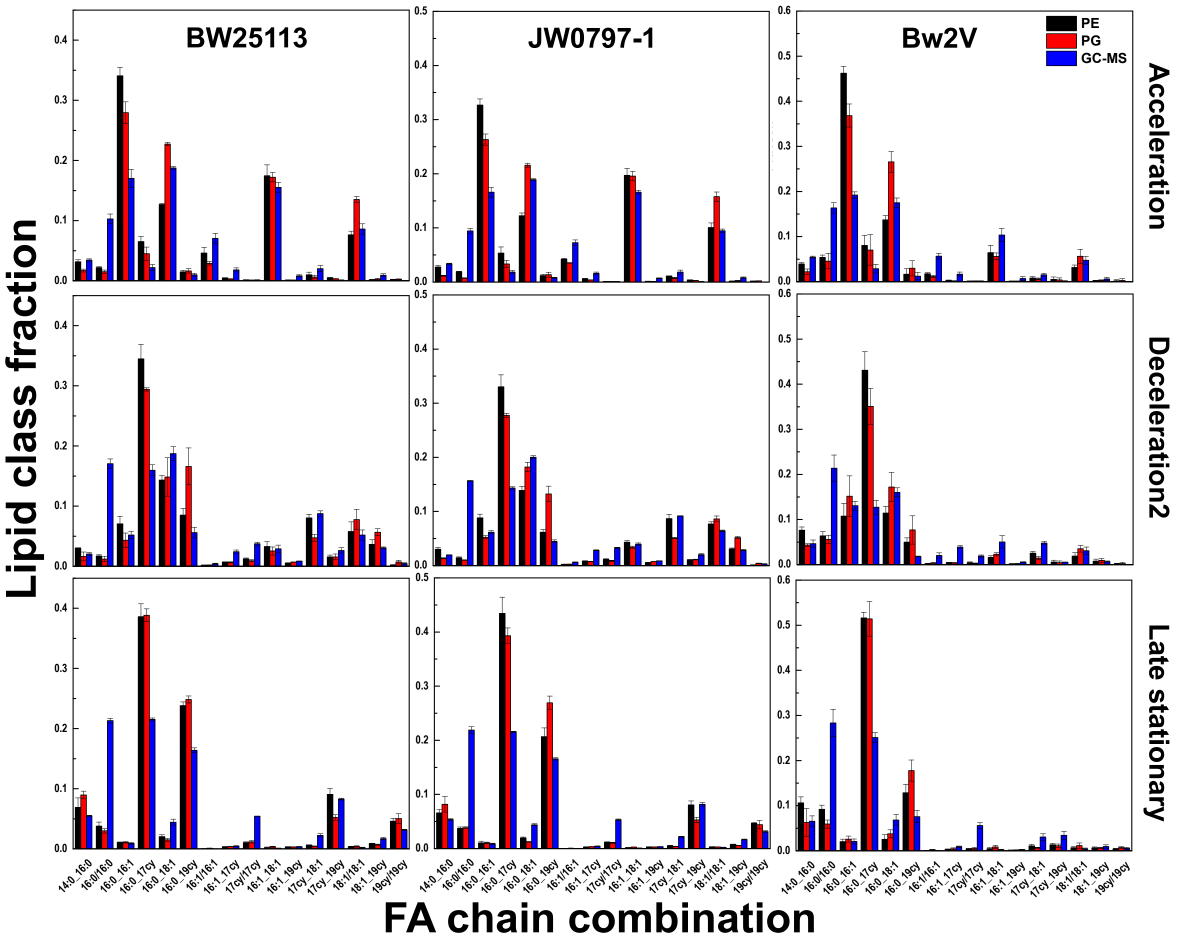

Figure S5. Non-random pairing of FA chains in PE and PG molecular species. The abundance of PE and PG molecular species from **Figure S3** differs from the random pairing of FA chains based on abundance. PE and PG molecular species abundance was determined using HILIC-MS[2]. Abundance of FA chains was determined using GC-MS, and the abundance of random pairs was estimated as their product. For example, the abundance of 16:0_16:1 was calculated as 2 × FA 16:0 × FA 16:1, where factor of 2 accounts for both *sn*-isomers. The abundance of 16:1/16:1 was a square of abundance of FA 16:1.

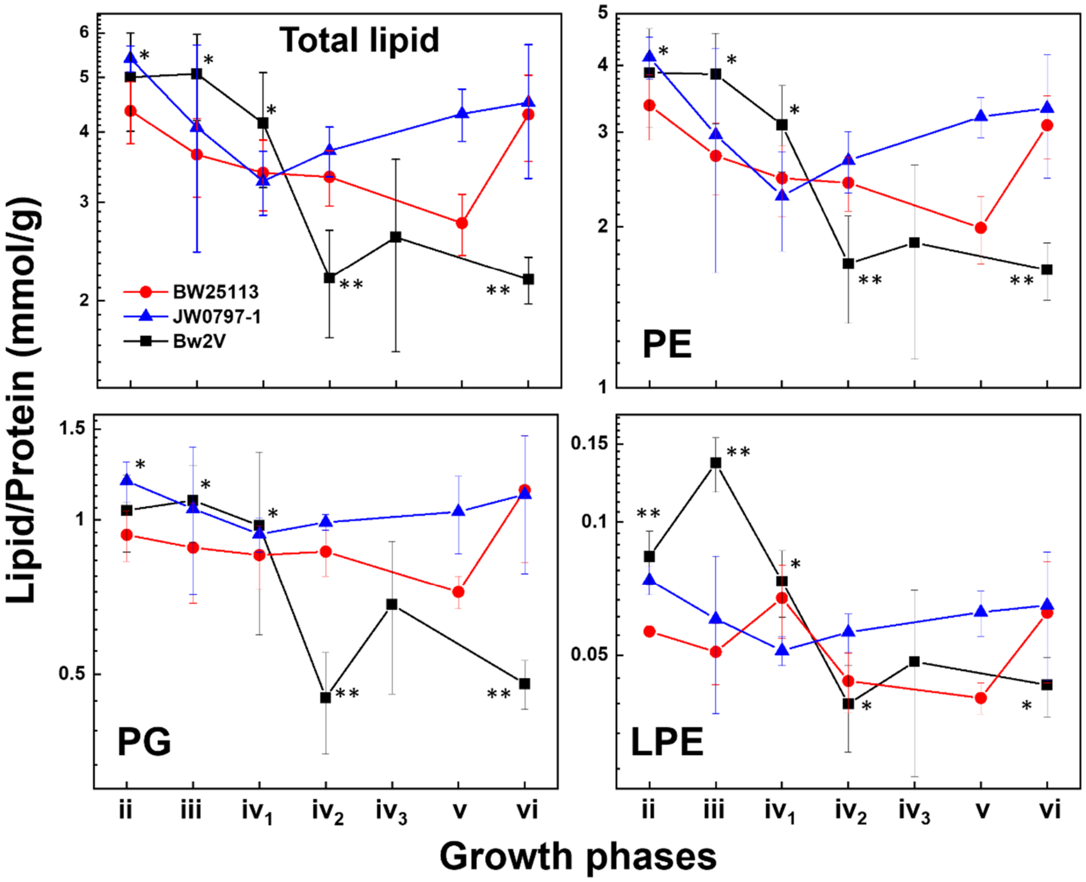

Figure S6. Total lipid and combined amounts of lipid classes of three *E. coli* strains in culture growth phases. * is for $p > 0.05$ using one way ANOVA, ** for $p < 0.05$ using two-tailed two-sample equal variance t-Test for BW25113 and Bw2V.
